# Marine-derived protein: peptide bioresources for the development of nutraceuticals for improved athletic performance

**DOI:** 10.3389/fspor.2023.1281397

**Published:** 2023-10-30

**Authors:** Mirza Hapsari Sakti Titis Penggalih, Ghevira Naila Praditya, Chrisandi Yusuf Rizqiansyah, Astuti Setyawardani, Athaya Febriantyo Purnomo, Reza Achmad Maulana, William Ben Gunawan, Dionysius Subali, Rudy Kurniawan, Nelly Mayulu, Nurpudji Astuti Taslim, Hardinsyah Hardinsyah, Yosef Stefan Sutanto, Fahrul Nurkolis

**Affiliations:** ^1^ Department of Nutrition and Health, Universitas Gadjah Mada, Yogyakarta, Indonesia; ^2^ Faculty of Medicine, Universitas Brawijaya, Malang, Indonesia; ^3^ Medical Student of Faculty of Medicine, University of Jember-Soebandi Regional Hospital, Jember, Indonesia; ^4^ Internship Doctor, Kanjuruhan General Hospital, Malang, Indonesia; ^5^ Department of Oncology, University of Oxford, Oxford, United Kingdom; ^6^ Nutrition Science, Faculty of Public Health, Ahmad Dahlan Univetsity, Yogjakarta, Indonesia; ^7^ Alumnus of Nutrition Science, Faculty of Medicine, Diponegoro University, Semarang, Indonesia; ^8^ Department of Biotechnology, Faculty of Biotechnology, Atma Jaya Catholic University of Indonesia, Jakarta, Indonesia; ^9^ Diabetes Connection Care, Eka Hospital Bumi Serpong Damai, Tangerang, Indonesia; ^10^ Department of Nutrition, Faculty of Health Science, Muhammadiyah Manado University, Manado, Indonesia; ^11^ Division of Clinical Nutrition, Department of Nutrition, Faculty of Medicine, Hasanuddin University, Makassar, Indonesia; ^12^ Division of Applied Nutrition, Department of Community Nutrition, Faculty of Human Ecology, IPB University, Bogor, Indonesia; ^13^ Department of Physical Medicine and Rehabilitation, Prof. R. D. Kandou General Hospital, Sam Ratulangi University, Manado, Indonesia; ^14^ Department of Biological Sciences, State Islamic University of Sunan Kalijaga (UIN Sunan Kalijaga), Yogyakarta, Indonesia

**Keywords:** sports food, marine protein, marine natural product, bioactive peptide, functional food, macronutrients, athletic performance

## 1. Introduction

Marine ecosystems, a prominent reservoir of biodiversity, have been globally acknowledged for their vast potential as sources of food and bioactive compounds. In recent years, an increasing number of studies have reported the discovery of novel proteins and peptides from marine organisms, thereby highlighting the untapped potential of these resources (1). These marine-derived proteins and peptides, owing to their unique amino acid composition, bioavailability, and bioactive properties, are being explored as a promising source of nutraceuticals and functional food ingredients (2) (Table 1).

**Table 1:** Marine-derived protein observed in several studies.

	Products/samples	Value/bioactivities	Reference
1	Antioxidant peptide Leu-Trp-His-Thr-His (LWHTH) from *Styela clava* (marine tunicate) (48)	ACE-Inhibitor	Kang et al. (2020)
2	Novel NCWPFQGVPLGFQAPP peptide (NCW peptide) from *Marphysa sanguinea* (marine polychaeta) (49)	antioxidant and anti-inflammatory	Park et al. (2020)
3	PFAOP peptide from *Pinctada fucata* (marine bivalve) (50)	Antioxidant	Ma et al. (2021)
4	HVGGCG peptide from *Oratosquilla woodmasoni* (marine squilla) (51)	ACE-Inhibitor and antioxidant	Joshi et al. (2021)
5	Gln-Trp-Arg Peptide from *Gadus chalcogrammus* (marine fish) (52)	Enhance glucose uptake to the muscle and lower blood glucose level	Ayabe et al. (2015)
6	Phe-Gly-Met-Pro-Leu-Asp-Arg (FGMPLDR; MW 834.41 Da) and Met-Glu-Leu-Val-Leu-Arg (MELVLR; MW 759.43 Da) peptide from *Ulva intestinalis* (microalgae) (53)	ACE-Inhibitor	Sun et al. (2019)
7	SFYYGK, RLVPVPY, and YIGNNPAKG peptide from *Gracilariopsis lemaneiformis* (marine red algae) (54)	ACE-Inhibitor	Su et al. (2022)
8	two phycobiliproteins (PBP): C-phycocyanin (C-PC) and allophycocyanin (APC) from *A. plantensis* (microalgae) (8)	Improve glucose metabolism	Karunarathne et al. (2020)
9	Val-Glu-Cys-Tyr-Gly-Pro-Asn-Arg-Pro-Gln-Phe (chlorella-11) from *C. vulgaris* and Leu-Asn-Gly-Asp-Val-Trp from *C. ellpsiodea* (microalgae) (55)	anti-inflamatory, blood glucose regulator	Ramos-Romero et al. (2021)
10	Skipjack Enzymatic Peptide (SEP) from *Katsuwonus pelamis* (marine fish) (56)	Anti-inflammatory	Wang et al. (2019)
11	DPP-IV inhibitor peptide from *Phaeodactylum tricornutum* and *Porphyridium purpureum* (microalgae) (57)	Antioxidant and antidiabetic	Stack et al. (2018)
12	DPPH-Scavenging peptides from *Dunaliella salina* (microalgae) (58)	Provitamin A, antioxidant, and food suplement for athlete diet	Çelebi et al. (2021)
13	Astaxanthin, carotenoids, protein, lutein, and fatty acid from *Haematococcus pluvialis* (microalgae) (59)	Anti-inflammatory, antioxidant, heal muscle soreness	Oslan et al. (2021)
14	GIISHR peptide from *Mustelus griseus* (Marine fish) (60)	Antioxidant	Ahmadi-Vavsari F et al. (2019)
15.	*I. galbana* peptide from *Isochrysis galbana* (microalgae) (61)	Anti-inflammatory	Bonfanti et al. (2018)
16	Marine peptide hydrolysate from salmon fish (62)	Metabolic influences during endurance cycling	Siegler et al. (2013)
17	Sardine scale peptide (63)	Improvement of the speed and strength indicators of the athletes from the test group and acceleration of recovery of the athletes after physical training	Mezenova et al. (2021)

Functional foods, or nutraceuticals, are dietary elements with added ingredients that offer health benefits beyond basic nutrition. They serve to enhance overall health, boost the immune system, reduce the risk of illness, and manage health conditions. Among the array of potential ingredients for functional foods, peptides have garnered significant attention. Peptides are short chains of amino acids that can be designed to have specific physiological benefits based on their structure and composition. They have been observed to possess various bioactive properties, such as antioxidant, antimicrobial, and anti-inflammatory activities, which potentially contribute to human health and wellbeing (3, 4).

The rapidly growing interest in functional foods is mirrored in the field of sports nutrition, where diet strategies aimed at optimizing athletic performance and recovery have become progressively more nuanced and specialized. Athletes continuously seek innovative dietary strategies that can safely improve performance, enhance recovery, and maintain overall health. The development of functional foods targeted at athletes, thus, represents a significant area of potential growth and research. Current trends in this field include the use of natural and sustainable sources of proteins and peptides, personalized nutrition strategies, and a focus on enhancing both physical and mental aspects of performance (5).

Despite the promising potential of marine-derived proteins and peptides in the development of nutraceuticals, there are still significant gaps in our understanding. The bioactivity of these compounds is influenced by several factors including their source, extraction methods, and the individual's physiological response, all of which need to be comprehensively understood to maximize their benefits. Moreover, the specific applications of these marine-derived proteins and peptides in sports nutrition are relatively unexplored. Athletes have historically harnessed the nutritional advantages of various marine products to enhance their performance and recovery. Among these, fish-based products have been particularly popular. Fish oil supplements, rich in omega-3 fatty acids, have been widely used to reduce inflammation, improve joint health, and support cardiovascular function in athletes. Additionally, marine protein supplements, often derived from sources like fish and shellfish, offer a concentrated source of essential amino acids, aiding in muscle repair and growth. Marine collagen supplements, obtained from fish scales and skin, have gained traction for their potential to enhance joint and connective tissue health, crucial for athletes engaging in high-impact activities. Furthermore, certain marine algae, such as spirulina and chlorella, have gained attention for their nutrient density, including protein content, making them suitable additions to athletes' diets for improved energy and recovery. These marine-derived products exemplify the diverse range of options available to athletes seeking to optimize their nutritional intake and gain a competitive edge (6).

The aim of this article is to review the potential of marine-derived proteins and peptides as a novel source of nutraceuticals, with a specific focus on their application in enhancing athletic performance. We strive to elucidate the current knowledge regarding their bioactive properties, discuss the challenges in their extraction and utilization, and explore the potential pathways for their incorporation into functional foods aimed at athletes. Ultimately, we hope to contribute to the broader understanding of marine bioresources and their role in the future of sports nutrition.

## 2. Marine natural product as bioresources in foods term

**Figure 1:**
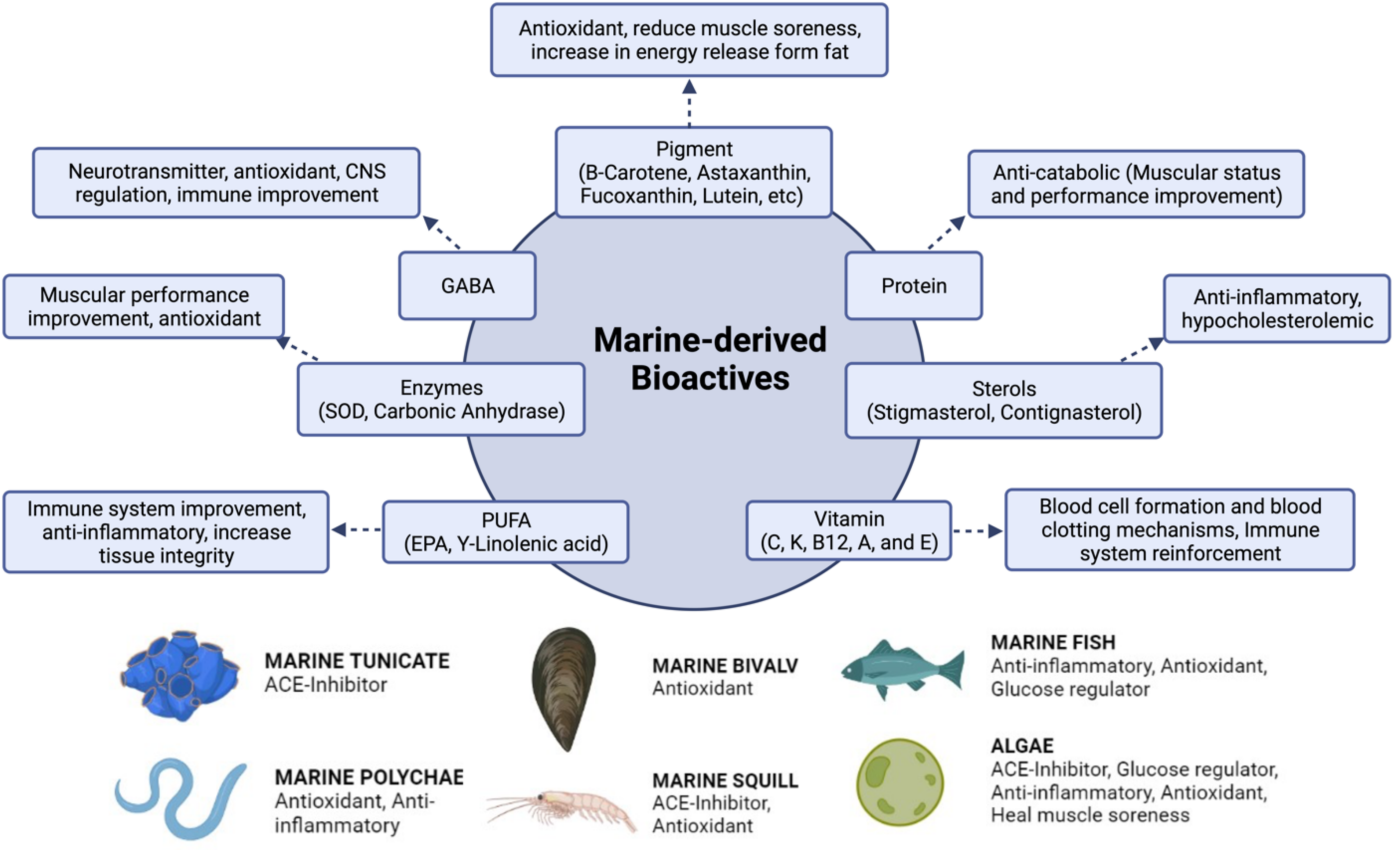
Marine organisms and their bioactive properties.

The marine environment is a reservoir of various natural products that have been widely used for medicine and beauty supplements, and have become a source of creation of new functional foods and nutritional supplements (1, 7). Carbohydrates, polyphenols, peptides, proteins, pigments, and essential fatty acids are examples of bioactive compounds obtained from various types of marine organisms, such as prokaryotes, algae, crustaceans, and other invertebrates, as well as various vertebrates (8, 9). Marine organisms have developed a wide range of bioactive chemicals that are not found in other organisms due to the diversity of their complex living conditions that give them a unique way of survival to grow and reproduce (8, 10). One of the widely used bioactive compounds is marine-derived peptides. Marine organisms that are well known for their peptide benefits are tunicates, fishes, seaweed, and various microorganisms (1). Here are some marine natural products as bioresources in food terms (Figure 1).

## 3. Marine-derived molecules and their nutritional values

In the past few years, functional and bioactive compounds from marine organisms such as sponges, bacteria, mollusks, and algae have been shown to have beneficial effects on health and could potentially be applied in medical activities (11). Unique bioactive compounds found in marine organisms, for example, peptides, polysaccharides, and fatty acids (12). Peptides from marine organisms are involved in the fundamental mechanisms that allow organisms to sustain life, including their reproduction, growth, and defense (13). The method for producing marine bioactive peptides is by solvent extraction or microbial protein fermentation which produces fragments with 3–20 amino acid residues (11). Marine-based purified peptide was found to exhibit potent ACE Inhibitor activity (12). Peptides derived from seaweed have shown potential to prevent cardiovascular disease and diabetes (14). Bioactive peptides sourced from fish are proposed to have an impact on the pathways that play a role in controlling blood pressure, as well as in regulating lipid and glucose metabolism and body composition (13). Peptides are also a promising alternative to antibiotics, such as peptide extracted from *Mytilus coruscus* (15).

Furthermore, bioactive peptides from marine microorganisms are starting to be applied as part of athlete's diet (16). Bioactive peptides were found to have a positive effect on body composition, namely increasing lean body mass and decreasing fat mass (17). Other effects include increasing muscle strength (17, 18), enhancing glucose intake into muscles (19), helping to heal muscle soreness and recovery from heavy exercise (20), and increasing the amount of upregulated proteins (myosin proteins, actin-binding proteins and tropomyosins) associated with resistance exercise adaptations (21). In addition, bioactive peptides have also been found to increase the translocation of GLUT-4 and GLUT-1 glucose transporters from the cytoplasm to the plasma membrane (22) which can have an impact on the enhancement of muscle glycogen and provide anti-stress effect (23). The ACE-inhibitory effect of bioactive peptides has also been found to improve endothelial function which is potentially beneficial for endurance sports (24, 25). Moreover, plasma biomarkers for muscle damage and inflammation were found to be lower in the group with bioactive peptide supplementation which shows that bioactive peptides can accelerate musculoskeletal adaptation and recovery through the possibility of extracellular matrix remodeling (26, 27).

Branched-Chains Amino Acids (BCAAS) consisting of leucine, isoleucine, and valine as peptide forming products also have many benefits for muscles, such as stimulating the synthesis of muscle protein (28), increasing physical performance, muscle strength, and muscle mass (29), and limiting muscle damage resulting from exercise (30). BCAA supplementation has been proven to improve the performance of athletes. Cheng et al. found that the supplementation of the BCAA could enhance endurance performance in college runners (31). Meanwhile, Chen et al. found that the supplementation of BCAA could alleviate the exercise-induced central fatigue in taekwondo athletes (32). In addition, leucine as a dietary supplement was also found to have an important therapeutic role in stress condition like burn, trauma, and sepsis, and also useful in slowing the degradation of muscle tissue. Leucine was found very high in various type of fishes, such as *S. Waitei*, *R. Kanagurta*, *L. Rohita*, *C. Mrigala*, *C. Batrachus* and *H. Fossilis*. Isoleucine is found in *O. Mykiss* and *L. Rohita* (33). Other studies found that leucine, isoleucine, and valine were contained in various other marine products such as tunas, mackerels, emperor fish, silky shark, and crustaceans such as lobsters and crabs. A serving of fish is found to provide approximately around or above 100% of the daily amounts of other essential amino acids recommended by the FAO and WHO, and a serving of crustaceans from the Palinuridae (spiny lobster) and Raninidae (spanner crab) families can be found to cover 60- 67% of valine, leucine, and isoleucine (34).

Marine products have the potential to be a source of ergogenic aids. Fish dan algae contain abundant beta alanine, creatine, and hydroxymethylbutyrate (HMB) that can improve the performances of athletes (35, 36). Beta alanine was proven to Increase time to exhaustion in athletes and increasing power output during strength training (37, 38). Creatine supplement was found to be able to delay fatigue at the time of exercise (39). Supplementing with HMB in athletes offers several benefits, including a favorable decrease in body fat while increasing lean muscle mass, enhancing anaerobic peak power, average power, and reducing post-anaerobic exercise lactate levels. Additionally, it helps limit the elevation of stress hormone response, preventing overreaching (40, 41).

Marine-derived antioxidants could also improve athlete performance and immune function by inhibiting the formation of muscle oxidative stress (42). Attenuation of oxidative stress found in young soccer athletes with antioxidant supplementation which was characterized by an increase in markers of lipid peroxidation malondialdehyde and total lipid peroxidation as well as a decrease in the ratio of glutathione to oxidized glutathione (43). Athletes who train at high altitudes also benefit from antioxidant supplementation, namely by reducing deformation of red blood cells (44). Reduced recovery period and delay of fatigue were also found when administering antioxidants immediately before and during exercise (45).

ACE-inhibitor content found in marine products also provides benefits for athlete performance. When exercising, the heart rate will increase to circulate blood throughout the body. An increase in heart rate will cause an increase in blood pressure, called autoregulation. The components of marine peptides have the opportunity to act as ACE inhibitors which work by inhibiting the enzyme that converts angiotensinogen to angiotensin (12). This will help control blood pressure. This condition causes dilation of blood vessels and a decrease in blood pressure. Marine peptide compounds help the heart work more efficiently and with less effort. This can reduce the risk of overworking the heart when athletes do intensive exercise. During intense exercise, blood pressure can increase significantly due to the increased oxygen demand by the muscles. This is the body's normal response to the physical load exerted during exercise. In addition to helping the heart work more efficiently, the opportunity of ACE inhibitors on marine peptides can provide additional protection for the kidneys. This effect can help reduce pressure on the glomerulus and the athlete's kidneys do not do extra work.

After exercise, there is a gradual decrease in blood pressure to normal levels or even below. This is caused by the release of blood vessel relaxing hormones, such as nitric oxide, which helps blood vessels widen and allows better blood flow (46). Marine's peptide compound has the opportunity to become an alternative food for athletes. Although there is research that currently states that the use of ACE inhibitor drugs has a non-synergistic effect on athletes, the use of natural ingredients in the form of Marine peptide compounds has not been studied further (47).

## 4. Future implication and direction in nutraceutical application for athletes

**Figure 2:**
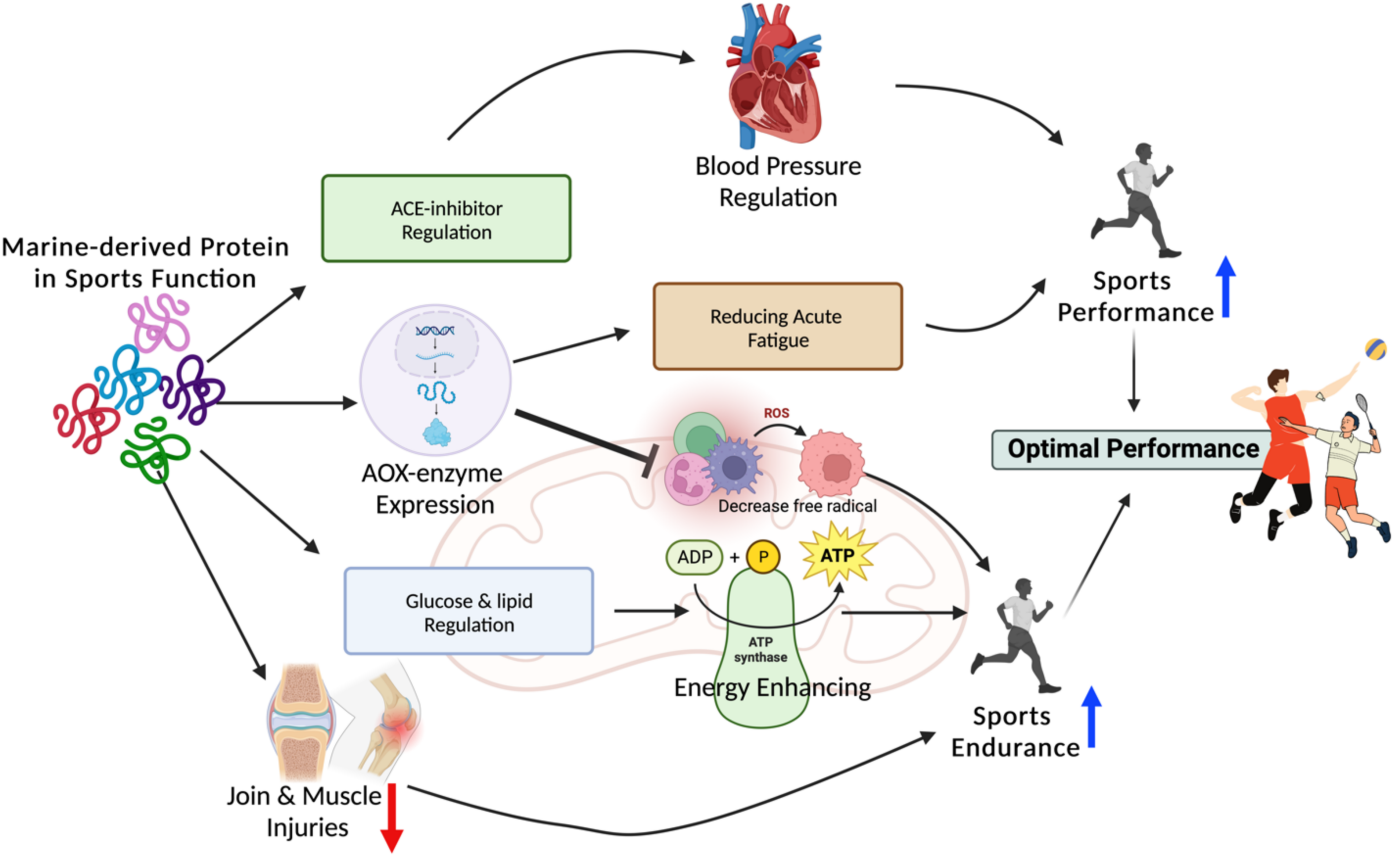
Marine-derived protein has beneficial effect to enhance sport performance and endurance by influence (1) ACE-inhibitor regulation affect to blood pressure regulation; (2) Antioxidant-enzyme expression affect to reducing acute fatigue and suppress reactive oxygen species (ROS) production; (3) glucose & lipid regulation affect to energy enhancing; and (4) decrease joint and muscle Marine-derived protein has beneficial effect to enhance sport performance and endurance by influence (1) ACE-inhibitor regulation affect to blood pressure regulation; (2) Antioxidant-enzyme expression affect to reducing acute fatigue and suppress reactive oxygen species (ROS) production; (3) glucose & lipid regulation affect to energy enhancing; and (4) decrease joint and muscle injuries.

Advanced nutritional interventions are one of the main subjects of elite sports performance globally. Moderate to high intensity sports require a high percentage of muscle mass with minimum body weight to generate the maximum power. Nutraceutical foods can be useful, to prevent and treat athletes' typical ailments, also improving their performance (64). Some negative physiological changes occur in long-lasting heavy training with immune system disturbance, inflammation, and stress oxidative could be deprived. Athletes and coaches ought to conduct thorough assessments tailored to each athlete's unique needs. To do so, they can delve into scientific information, focusing on essential aspects. For instance, in-depth analysis of human physiological fluids like blood, urine, and feces can provide valuable insights into dietary necessities and nutritional objectives. This information can then inform the selection of appropriate medical supplements and sports nutrition. These personalized dietary plans can be customized to cater to an athlete's requirements through various means, such as consolidating multiple nutrients into a single delivery method or integrating diverse delivery systems containing different nutrients (Figure 2) (65, 66).

In recent years, there has been a growing interest in the potential of marine-derived substances to combat obesity-related health issues, such as dyslipidemia, diabetes, oxidative stress, and inflammation. These bioactive compounds have shown promising effects in addressing these conditions and have thus become a focus of research and development. Marine-based products, known for their abundance of natural bioactive molecules like omega-3 fatty acids, proteins, biopeptides, carotenoids, glucosamine, and minerals, have the potential to be developed into a valuable source of nutritional food for athletes. These products offer a range of benefits, including enhanced performance, improved recovery, and overall support for the unique nutritional needs of individuals engaged in intense physical activity (64). Recent studies have shown that omega-3 fatty acids, found in marine-based products, can have a significant impact on the metabolic and functional responses of skeletal muscle during exercise training. These fatty acids not only offer potential anti-inflammatory and antioxidant benefits but also contribute to faster cell regeneration, aiding in the recovery process for athletes (64–67). Another example is positive impact on muscular performance and reduced muscle damage found after administration of bioactive peptides (68). This shows that as a nutritional source that is environmentally friendly and has a diverse product offering, as well as possessing many unique nutrients that are not often found in traditional sports supplements, marine-derived products could be a sustainable source of supplements for athletes and able to compete effectively with established commercial athletic products. Currently, there is a lack of knowledge regarding the suitable type and concentration of various bioactive components for specific individuals. As a result, it is anticipated that in the coming years, new formulations will be developed, considering potential benefits over traditional ones and advancements in oral bioavailability. These advancements may involve the use of innovative techniques to enhance the delivery and effectiveness of marine-based bioactive compounds. Further studies were needed focusing on marine-derived protein development and functional food manufacturing for high-performance athletes. Practical forms in a combination of marine-derived protein with daily dietary intake or as a dietary supplementation were expected. This article calls on researchers to promote marine-derived bioactivities, especially in the athlete population.
